# Elevated Sodium and Dehydration Stimulate Inflammatory Signaling in Endothelial Cells and Promote Atherosclerosis

**DOI:** 10.1371/journal.pone.0128870

**Published:** 2015-06-04

**Authors:** Natalia I. Dmitrieva, Maurice B. Burg

**Affiliations:** National Heart, Lung, and Blood Institute, National Institutes of Health, Bethesda, Maryland, United States of America; University of Milan, ITALY

## Abstract

Cardiovascular diseases (CVDs) are a leading health problem worldwide. Epidemiologic studies link high salt intake and conditions predisposing to dehydration such as low water intake, diabetes and old age to increased risk of CVD. Previously, we demonstrated that elevation of extracellular sodium, which is a common consequence of these conditions, stimulates production by endothelial cells of clotting initiator, von Willebrand Factor, increases its level in blood and promotes thrombogenesis. In present study, by PCR array, using human umbilical vein endothelial cells (HUVECs), we analyzed the effect of high NaCl on 84 genes related to endothelial cell biology. The analysis showed that the affected genes regulate many aspects of endothelial cell biology including cell adhesion, proliferation, leukocyte and lymphocyte activation, coagulation, angiogenesis and inflammatory response. The genes whose expression increased the most were adhesion molecules VCAM1 and E-selectin and the chemoattractant MCP-1. These are key participants in the leukocyte adhesion and transmigration that play a major role in the inflammation and pathophysiology of CVD, including atherosclerosis. Indeed, high NaCl increased adhesion of mononuclear cells and their transmigration through HUVECs monolayers. In mice, mild water restriction that elevates serum sodium by 5 mmol/l, increased VCAM1, E-selectin and MCP-1 expression in mouse tissues, accelerated atherosclerotic plaque formation in aortic root and caused thickening or walls of coronary arteries. Multivariable linear regression analysis of clinical data from the Atherosclerosis Risk in Communities Study (n=12779) demonstrated that serum sodium is a significant predictor of 10 Years Risk of coronary heart disease. These findings indicate that elevation of extracellular sodium within the physiological range is accompanied by vascular changes that facilitate development of CVD. The findings bring attention to serum sodium as a risk factor for CVDs and give additional support to recommendations for dietary salt restriction and adequate water intake as preventives of CVD.

## Introduction

Cardiovascular diseases (CVDs) are a leading health problem worldwide.[1, 2] Epidemiologic studies link high salt intake and conditions predisposing to dehydration, such as low water intake, diabetes and old age to increased risk of CVD [3–7]. The underlying mechanisms are not fully understood. A common consequence of these conditions is elevation of plasma sodium. [4, 8–12] Sodium (Na^+^) and chloride (Cl^-^) are the major electrolytes in plasma and extracellular fluids. Their concentration is maintained within narrow range by osmoregulation. Normal plasma sodium concentration in the general population varies between 134 and 148 mmol/l. [13–15] During hypernatremia plasma sodium concentration increases beyond the normal range, and it can reach 160 mmol/l or higher in cases of severe life threatening hypernatremia [12]. Elevation of plasma sodium within or above the normal range is a common consequence of dehydration [9, 12], high salt consumption [10, 16], and conditions affecting water/salt balance [8, 11, 12, 17]. Since all of these conditions are associated with increased risk of CVDs [3–7, 18], we hypothesized that direct effects of elevated sodium on endothelium might be a contributing factor.

The known mechanisms underlying the pathology of cardiovascular diseases such as atherosclerosis, coronary heart disease (CHD) and stroke involve a complex interplay of inflammation and coagulation [19–21] of which endothelial cells are major regulators. Resting endothelial cells inhibit thrombosis and inflammation by releasing anti-coagulants and suppressing release of pro-coagulants and inflammatory mediators. Endothelial activation, leading to release of mediators of coagulation and inflammation and increased adhesiveness of leukocytes, is implicated in pathophysiology of CVD.[22, 23]

There are indications that minor elevations of extracellular sodium may have adverse effects, leading to clinically relevant consequences. Thus, increase in the sodium concentration of the culture medium within physiological range stiffens endothelial cells, reduces nitric oxide release [24, 25] and leads to hardening and flattering of endothelial glycocalyx (eGC) [25] which is a major modulator of endothelial cells functions. [26] High sodium also potentiates pro-inflammatory effects of TNFα and of non-uniform shear stress.[27] Our recent finding that minor elevations of extracellular sodium upregulate expression and secretion of a key initiator of blood clotting, von Willebrand Factor (vWF) in endothelial cells also supports this hypothesis [28]. It has been recently shown that plasma sodium does not vary randomly, but is characteristic of individuals that depends on long-term life style, health status or genetically defined factors [29], indicating that it could affect long-term health. Indeed, in multivariable regression analysis of clinical data from the Atherosclerosis Risk in Communities Study, serum sodium significantly contributes to prediction of the blood level of vWF and the 10 years Risk of Stroke [28]. In addition, it has been recently demonstrated that higher levels of plasma sodium are associated with higher risk of mortality in a healthy population.[14]

Here we report that small, physiological elevations of extracellular sodium in cell culture and mild dehydration that elevates plasma sodium in mice activate inflammatory signaling (increased VCAM-1, E-selectin and MCP-1 expression), increase adhesive properties of endothelial cells, and lead to vascular changes that promote atherosclerosis and thickening of walls of coronary arteries. Further, in humans, serum sodium is positively associated with risk of CHD. Taken together, our findings indicate that elevation of extracellular sodium, even within the physiological range, is accompanied by vascular changes that facilitate development of CVD.

## Results

### Elevation of extracellular NaCl upregulates pro-inflammatory mediators in endothelial cells in culture and increases their adhesive properties

**Fig 1:**
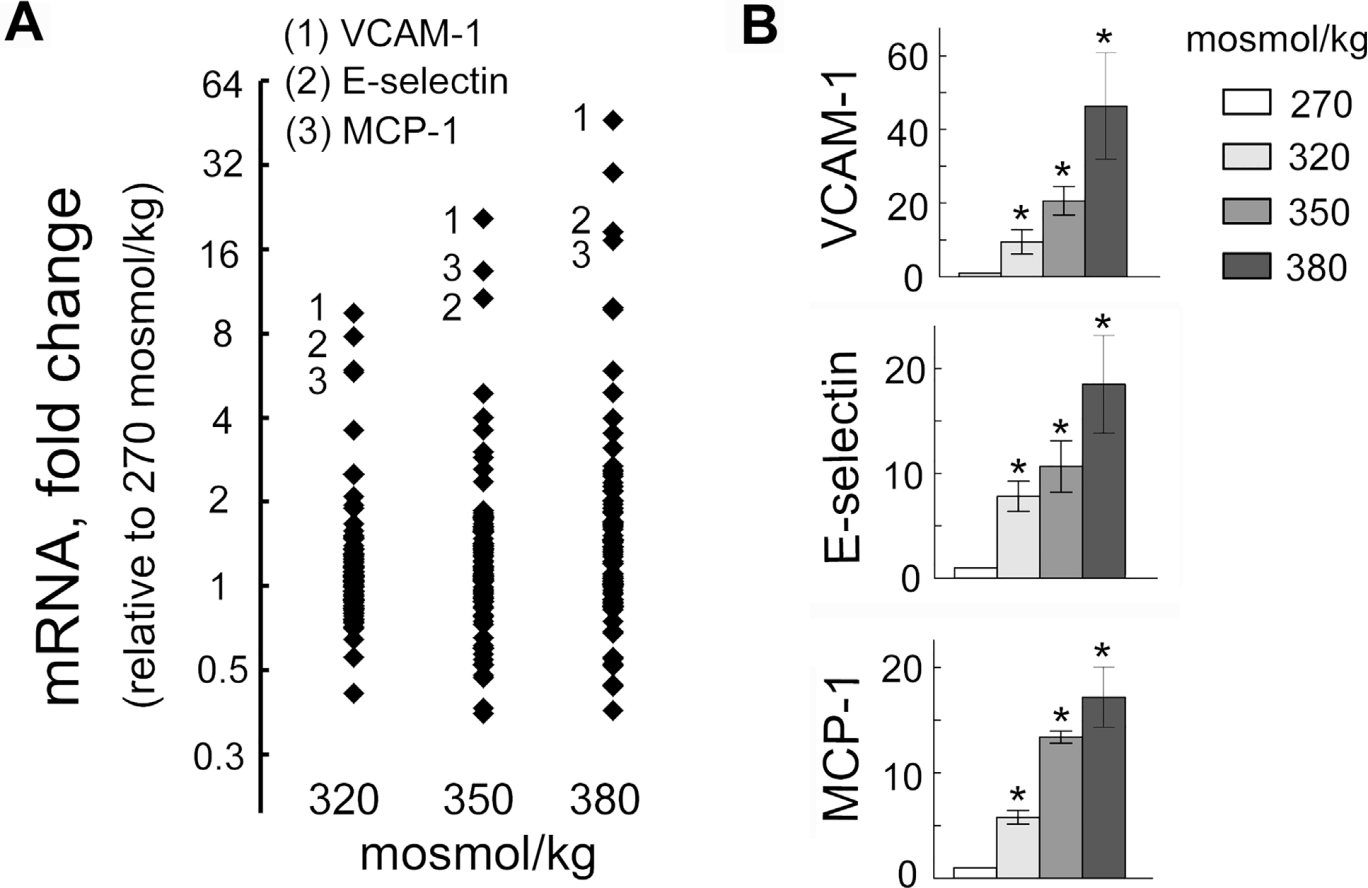
Elevation of extracellular NaCl increases expression of pro-inflammatory mediators in endothelial cells. HUVECs were exposed to media in which NaCl was elevated for 4 days from 270 mosmol/kg to the total osmolality indicated in the figure panels. mRNA for 84 genes was measured by real-time PCR as described in Methods. **(A)** mRNA of adhesion molecules VCAM-1, E-selectin and of chemokine MCP-1 showed biggest increase upon exposure to high NaCl. The graph plots fold changes relative to 270 mosmol/kg of mRNA for 84 genes related to endothelial cell biology. Each dot shows mean value from 3 independent experiments. **(B)** Statistical analysis for VCAM-1, E-selectin and MCP-1 (mean ±SEM, *P<0.05, N = 3)

To assess effect of elevated NaCl on endothelial cells, we used a PCR array to analyze the effect of high NaCl on 84 genes related to endothelial cell biology (Fig 1, Table 1). We exposed HUVEC cells grown on cell culture dishes to different levels of NaCl for 4 days. The genes whose expression increased or decreased at least two-fold are listed on Table 1. To get an initial insight to what functions of endothelial cells might be affected by changes in expression of these genes, we used the DAVID Gene Functional Classification Tool [30]. The analysis showed that the affected genes regulate many aspects of endothelial cell biology as well as their interaction with blood cells. Thus, changes in expression of those genes affects cell migration, cell adhesion, proliferation, leukocyte and lymphocyte activation, apoptosis, hemopoiesis, coagulation, angiogenesis and inflammatory response (Table 1). Since disregulation of these functions is often involved in pathological processes like inflammation, vascular wall remodeling and thrombosis [31], we next tested if high NaCl causes such functional consequences, focusing on inflammation. The three genes that showed biggest increase were the adhesion molecules VCAM-1 and E-selectin and the chemokine, MCP1 (Fig 1). These molecules are key players in leukocyte adhesion and transmigration, which are hallmarks of inflammation [32]. During their inflammatory activation, endothelial cells express the adhesion molecules on their plasma membrane and secrete chemokines and cytokines. MCP-1 is a chemokine that activates monocytes, facilitating their capture by adhesion molecules and their transmigration through the endothelium into tissues [32, 33].

**Table 1:** Effect of high NaCl on expression of genes related to endothelial cell function in cultured HUVEC cells.

Gene Symbol	Fold change (relative to 270 mosmol/kg)			Functional Annotations for the genes**										
	320 mosmol/kg	350 mosmol/kg	380 mosmol/kg	1	2	3	4	5	6	7	8	9	10	11
VCAM1	9.5*	20.6*	46.3*	✓	✓	✓	✓	✓	✓				✓	
SELE	7.8*	10.7*	18.5*	✓	✓	✓				✓				
CCL2	5.8*	13.4*	17.2*	✓	✓		✓			✓	✓			
BAX	2.5*	4*	30	✓				✓	✓		✓		✓	
IL11	2.5*	4.9*	9.9*				✓	✓	✓				✓	✓
FN1	2*	2.4*	2.6*	✓		✓				✓		✓		
RHOB	1.5	1.8*	2.6*			✓						✓		
IL7	1.3*	1.8*	2.5*				✓	✓	✓		✓		✓	
PLA2G4C	1.5	1.6	2.5*							✓				
FGF1	1.3	1.7*	2.3*				✓					✓		
VEGFA	0.8*	1.6*	2.3*				✓				✓	✓	✓	
OCLN	1.3*	1.3	2*											
VWF	1.1	1.4*	2*			✓								✓
CRADD	1.7*	1.9*	2*											
ITGB1	1.4	1.8*	2*	✓		✓	✓	✓	✓				✓	
KDR	0.9	0.5*	0.6*	✓			✓					✓	✓	
KLK3	0.8	0.5*	0.5*											
PF4	0.5*	0.6*	0.5	✓	✓						✓			✓
SPHK1	0.8*	0.7	0.5*				✓				✓			
RPL13A	0.8	0.5*	0.5*											
ADAM17	0.7*	0.5*	0.4*	✓			✓	✓	✓		✓		✓	
ACE	0.8	0.5*	0.4*										✓	

The Human Endothelial Cell Biology PCR Array was used to determine relative changes in expression of 84 genes caused by increasing NaCl in the cell culture medium. The table includes those genes whose expression increased or decreased significantly more than 2-fold following increase in extracellular NaCl and lists the functional gene ontology (GO) categories to which these genes belong.

*Change is significant with P < 0.05, n = 3

**Gene Ontology (GO) terms associated with the genes: 1) cell migration; 2) leukocyte migration; 3) cell adhesion; 4) stimulation of proliferation; 5) Leukocyte activation; 6) lymphocyte activation; 7) inflammatory response; 8) negative regulation of apoptosis; 9) angiogenesis; 10) hematopoiesis; 11) coagulation. The analysis was done with the DAVID Functional Annotation Tool.

**Fig 2:**
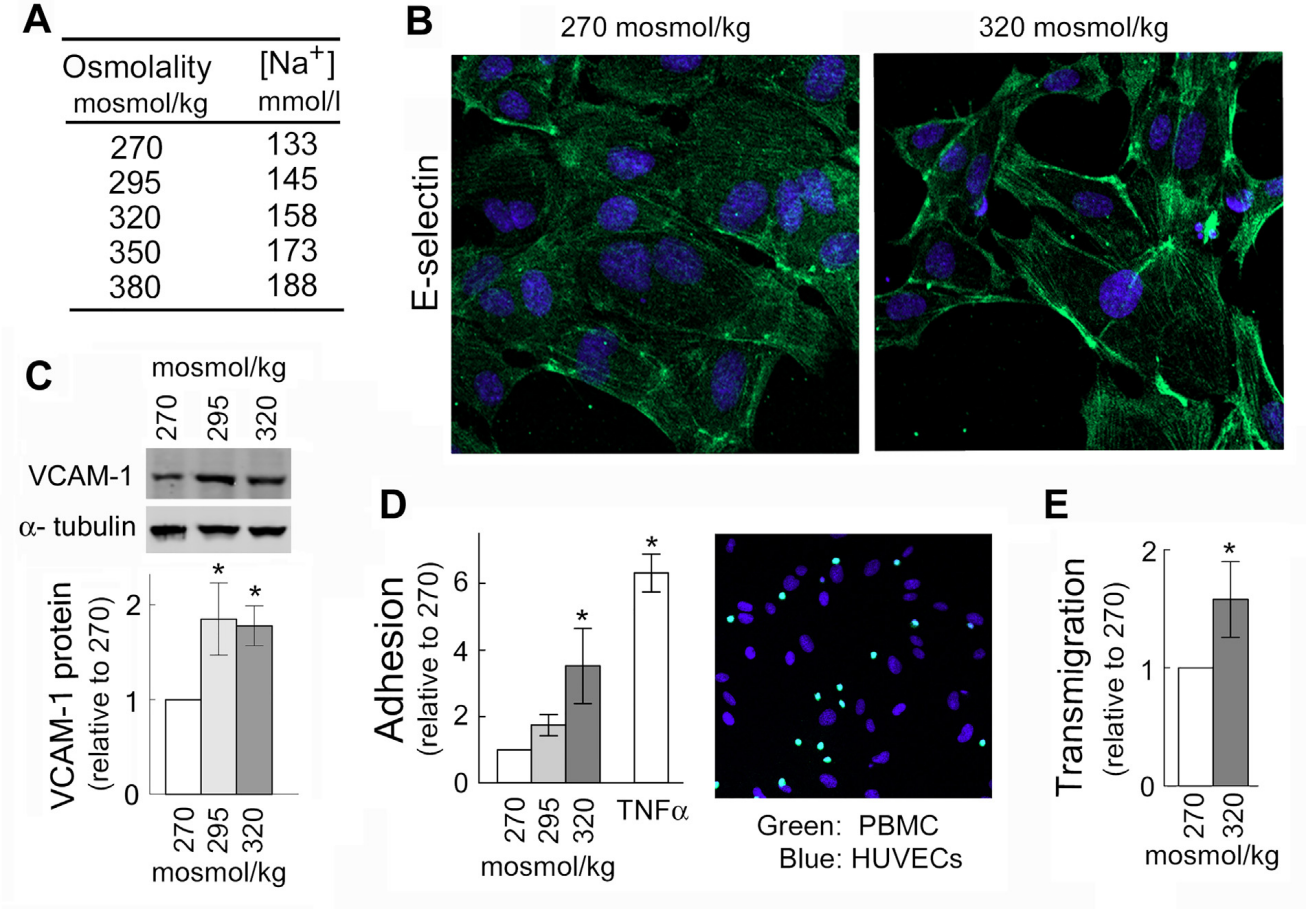
Elevation of NaCl in cell culture medium promotes adhesion of peripheral blood mononuclear cells (PBMC) to HUVECs and their transmigration through HUVEC monolayer. HUVECs were exposed to media in which NaCl was elevated for 4 days from 270 mosmol/kg to the total osmolality indicated in the figure panels. (A) The table shows sodium concentrations in culture medium of different osmolarities used in experiments shown on Figs 1 and 2. Note, that blood sodium range 135–145 mmol/l is considered normal; up to 160 mmol/l can be seen in people without symptoms (12); and higher levels are seen only in cases of severe hypernatremia in critically ill patients. (B) High NaCl increases E-selectin, a mediator for PBMC adhesion, in plasma membrane of HUVECs (representative IF image: green (Alexa488)—E-selectin, blue—cell nuclei stained with DAPI). (C) High NaCl increases VCAM-1 protein. *Upper Panel*: Representative Western Blot. *Lower Panel*: Quantification, relative to 270 mosmol/kg, normalized to tubulin (mean ±SEM, *P<0.05, t test, N = 3). (D) Elevation of NaCl within physiological range causes graded increase of adhesive properties of HUVECs. *Left panel*: Quantification of adhesion. Data are plotted as number of PBMC adhered to 1000 HUVECs (mean ±SEM, *P<0.05, N = 4, linearly dependent on NaCl concentration, P = 0.03). Note: Treatment with TNFα, known inducer of the adhesion was used as a positive control. *Right panel*: Representative image of stained cells used for the quantification of PBMC (green, calcein AM) attached to HUVECs (blue, DAPI). See methods for more details. (E) Exposure to elevated NaCl promotes transmigration of PBMC through HUVEC monolayer. Number of PBMC transmigrated in 6h was quantified relative to 270 mosmol/kg (mean ±SEM, *P<0.05, N = 3). See methods for more details.

To test whether high NaCl-induced expression of VCAM-1, E-selectin and MCP-1 affects adhesive properties of endothelial cells, we analyzed effects of high NaCl on adhesion of peripheral blood mononuclear cells (PBMC) to HUVECs and on their transmigration through HUVEC monolayers. In these experiments, we added NaCl to the cell culture medium to elevate sodium over a range of blood sodium from the lower end of the normal range (133 mmol/l) up to extreme hypernatremia (160 mmol/l) (Fig 2A). Indeed, consistent with the increased mRNA of the adhesion molecules (Fig 1), exposure of HUVECs to high NaCl elevates E-selectin protein in the plasma membrane (Fig 2B), increases VCAM-1 protein level (Fig 2C), increases adhesion of peripheral blood mononuclear cells (PBMC) to HUVECs (Fig 2D) and increases transmigration of PBMC through HUVEC monolayers (Fig 2E). These results indicate that elevation of extracellular NaCl over the range that occurs in humans increases the adhesive properties of endothelial cells suggesting that elevated NaCl can initiate inflammation.

### Water restriction activates pro-inflammatory signaling in tissues and accelerates atherosclerosis in mice

Having found that elevations of NaCl increase expression of VCAM-1, E-selectin and MCP-1, leading to increased adhesive properties of endothelial cells in culture, we next tested whether increase of serum sodium has similar effects in vivo.

**Fig 3:**
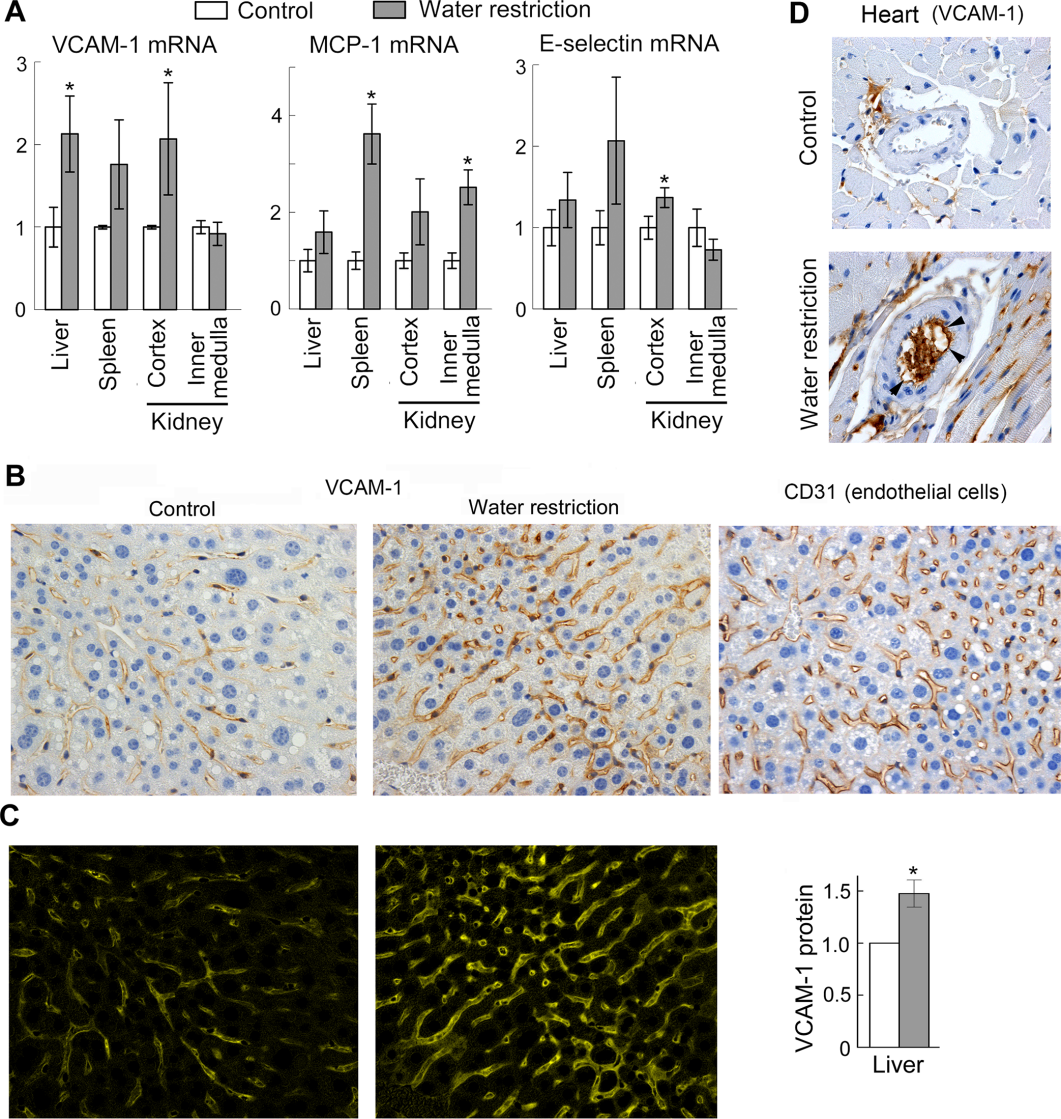
Water restriction activates pro-inflammatory signaling in mouse tissues. To elevate NaCl in vivo, mice were subjected to water restriction for 9 days. To limit the amount of water, mice were fed with gel food containing 30% water and were not given any additional water. Control group mice were fed the same gel food, but had free access to water. (A) Water restriction increases VCAM-1, MCP-1 and E-selectin mRNA in several tissues. Levels of the mRNA were measured by real-time PCR. Results are presented as mean ±SEM, *P<0.05, N = 5. (B, C) Water restriction increases VCAM-1 protein in endothelial cells in the liver. (B) Representative images from immunohistochemical staining for VCAM-1 protein and for endothelial cells marker CD31 (brown) in the liver tissue sections. Similar patterns of VCAM-1 and CD31 staining are consistent with endothelial expression of VCAM-1. (C) Quantification of VCAM-1 staining shown on B. See methods section for more information about the quantification. *Left panels*. Images of extracted CMYK yellow channel from images shown on B. The pattern and intensity of the signal in the CMYK yellow channel corresponds to pattern and intensity to the brown diaminobenzidine (DAB) staining of VCAM-1. *Right panel*. Result of the quantification (mean ±SEM, *P<0.05, N = 4). (D) Representative images from immunohistochemical staining for VCAM-1 protein of heart sections. Positive staining of inner surface of coronary arteries of water restricted mice (shown by arrowheads) is consistent with endothelial expression of VCAM1. See S1 Fig for more images.

We controlled the amount of water that the mice consumed by feeding them with gel food containing 30% water, as their only source of water intake or, as a control, feeding the same food, but with free access to drinking water [28]. We previously showed that such water restriction increases serum sodium by about 5 mmol/l but does not cause weight loss, indicating a very mild dehydration that could occur in everyday life. [28] Consistent with the cell culture results, the mild dehydration increases VCAM-1, E-selectin and MCP-1 mRNA in several tissues (Fig 3A). We next confirmed by immunohistochemical staining of the liver and heart sections that VCAM-1 protein expression is increased in endothelial cells of the liver capillaries (Fig 3B and 3C), and coronary arteries (Fig 3D, S1 Fig) suggesting that the mild dehydration might be pro-inflammatory.

**Fig 4:**
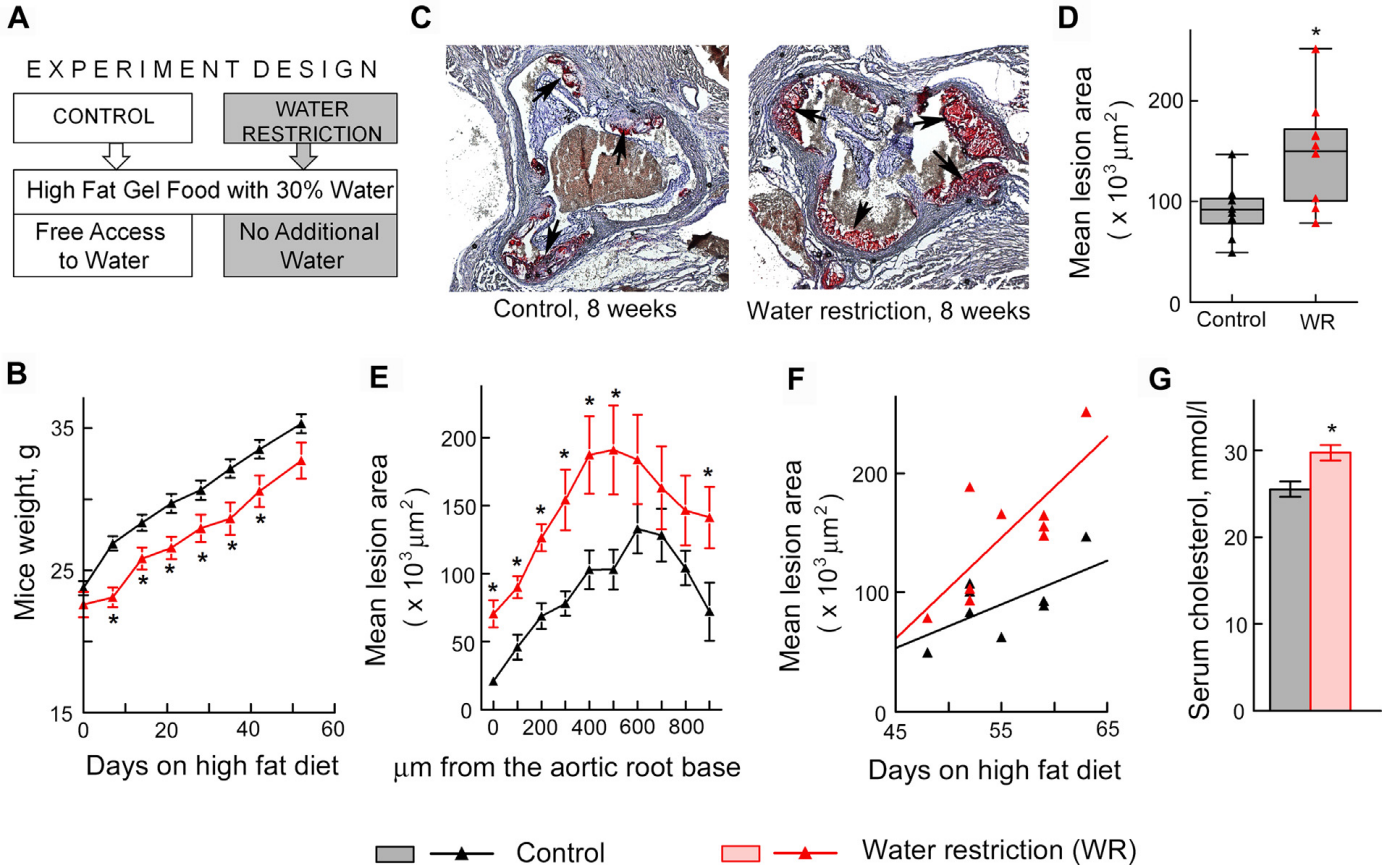
Water restriction accelerates atherosclerosis in ApoE^-/-^ mice. Mice were water restricted starting at 6 weeks of age for 7–9 weeks and atherosclerosis was analyzed in aortic root as described in methods. Data are presented as mean ±SEM, *P<0.05, N = 9. (A) Experiment design. Water restricted mice were fed with gel food made from “western” diet containing 40% of calories from fat and 30% water and were not given any additional water. Control group were fed the same gel food, but had free access to water. (B) Water restricted mice grow at the same rate as controls after transient growth retardation. (C-F) Atherosclerotic lesions are larger in water restricted mice. (C) Representative images of frozen sections through aortic root in which lipids in the atherosclerotic lesions are stained in red with Oil Red O dye (shown by arrows). (D) The graph pots mean areas of the lesions on serial sections spanning approx. 900 μm of the aortic root. The data are presented as a box-and-whisker plot (n = 9, t test, P = 0.01). (E) The graph plots mean areas of the lesions at different distances from the base of the aortic root. (F) Atherosclerotic lesions grow faster in water restricted mice as shown by steeper regression line on the scatterplot of mean lesion area vs time (see methods for more details). (G) Serum cholesterol was measured after 6 weeks on high fat diet and water restriction.

**Fig 5:**
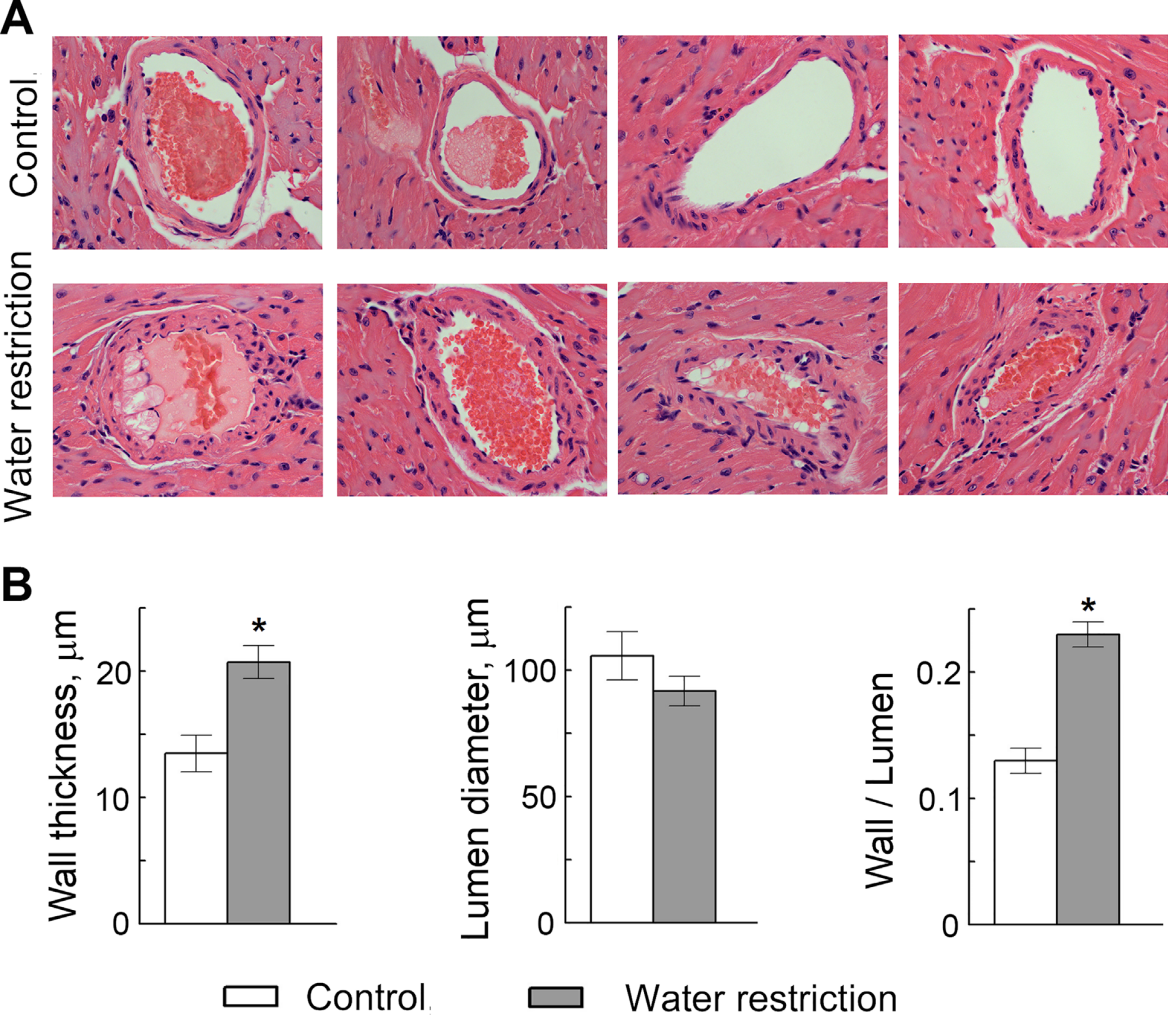
Water restriction causes thickening of the walls of coronary arteries in ApoE^-/-^ mice. (A) Representative images from hematoxylin and eosin staining of heart sections showing coronary arteries. (B) Quantification of the arterial wall thickness, lumen diameter and wall thickness to lumen diameter ratio. Data are presented as mean ±SEM, *P<0.05, N = 7.

Since VCAM-1-facilitated recruitment of leukocytes from the circulation is an initial step in many inflammatory disorders, including atherosclerosis. [34–37], we hypothesized that water restriction might affect formation of atherosclerotic plaques. To test the effect of water restriction on development of atherosclerosis, we used apolipoprotein E knockout (ApoE^-/-^) mice. ApoE^-/-^ mice have impaired clearance of plasma lipoproteins and develop atherosclerosis in a short time, making them a convenient model to assess impact of different factors. [38] We fed ApoE^-/-^ mice a “western” diet containing 40% of calories from fat and analyzed effect of water restriction on size of atherosclerotic lesions in the aortic root (Fig 4). [39] To control water intake, we fed mice with gel food made from the “western” diet and containing 30% of water. Control mice had free access to water (Fig 4A). Such water restriction did not cause severe dehydration as judged from the weight of the mice. After transient growth retardation that lasted about 1 week, water restricted mice resumed growth at the same rate as the control group (Fig 4B). We analyzed atherosclerotic lesions in the aortic root after 7–9 weeks of water restriction. Consistent with our hypothesis that sodium-induced stimulation of inflammatory signaling and of the adhesive properties of the endothelium might accelerate atherosclerosis, water restricted mice developed larger atherosclerotic lesions in the aortic root (Fig 4C–4F). Water restriction also caused slight but significant increase in serum cholesterol (Fig 4G), which might be an additional factor contributing to the acceleration of development of atherosclerosis in water restricted ApoE^-/-^ mice. [40] Additionally, water restriction caused thickening of the walls of coronary arteries in the ApoE^-/-^ mice (Fig 5), which is considered as a risk factor for development of atherosclerosis and predicts future clinical cardiovascular disease events.[41, 42]

### Plasma sodium is positively associated with 10 Years Risk of coronary heart disease (CHD) in Atherosclerosis Risk in Community (ARIC) Study

In order to assess the relevance of our findings to humans, we analyzed whether there is an association between plasma sodium concentration and the risk of CHD, using data from the Atherosclerosis Risk in Communities (ARIC) Study. ARIC is a study of cardiovascular disease in a cohort of 15792 45- to 64-year-old persons sampled from four US communities in 1987–1989 [43]. We obtained the ARIC study data from the NHLBI Biologic Specimen and Data Repository Information Coordinating Center (BioLINCC). We used the measurements of serum sodium and blood glucose obtained during baseline clinical examination of the participants on their first visit, and 10 Year Risk of CHD at first visit. The 10 Years Risk of CHD at first visit was retrospectively calculated for participants in the ARIC study based on the study outcomes and included in the ARIC datasets.[44] It can vary between 0 to 100%. Participants were separated based on gender, race, and diabetic status. The 10 years risk of CHD was then calculated using the Cox regression equation. The beta-coefficients used for the prediction for people with and without diabetes were previously published in ARIC manuscripts. [44, 45] The risk factors that were initially discovered by the Framingham Heart Study and confirmed by the ARIC Study, used to calculate risk scores were total cholesterol, high density cholesterol, blood pressure, medications for hypertension and smoking [44–46]. The beta-coefficients for these risk factors were estimated based on the following definition of CHD events: a CHD event was defined as a validated definite or probable hospitalized MI, a definite CHD death, an unrecognized MI defined by ARIC ECG readings, or coronary revascularization. The criteria for definite or probable hospitalized MI were based on combination of chest pain symptoms, ECG changes, and cardiac enzyme levels [44, 47]

**Fig 6:**
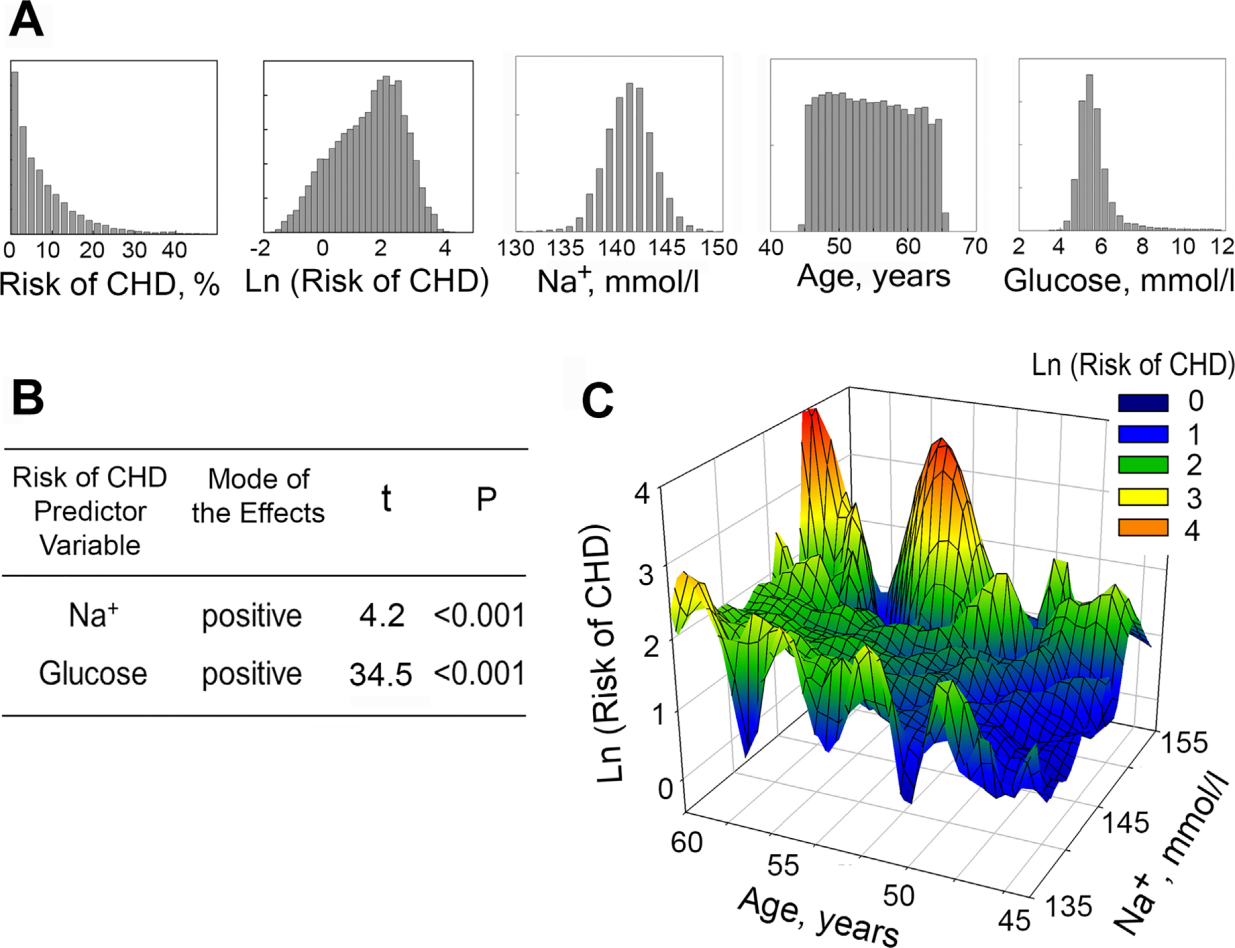
Plasma sodium is positively associated with 10 Years Risk of CHD in Atherosclerosis Risk in Community (ARIC) Study. (A, B) Multivariable linear regression analysis was used to assess effect of serum sodium on 10 Years Risk of CHD (N = 12779). (A) Distribution histograms for variables used in the analysis. (B) Overview of the results. The results demonstrate that serum Na^**+**^, as well as glucose, significantly contributes to predicting the Risk of CHD. See text and Table 2 for details of the analysis. (C) 3D Mesh Plots, visualizing Ln(10 Years Risk of CHD), as functions of serum sodium concentration and age. At all ages, 10 Years Risk of CHD is increased in participants with higher levels of serum sodium.

We reasoned that the estimated CHD Risk serves as an approximate measure of atherosclerosis burden, and we hypothesized that in line with the results of the mouse studies, plasma sodium might affect atherosclerosis and therefore the risk of CHD. To assay if the concentration of serum sodium contributes to prediction of 10 Year Risk of CHD, we conducted multivariable linear regression analysis. [48–51] The following variables from ARIC study were used: serum sodium (mmol/l), blood glucose (mmol/l) and 10 Year Risk of CHD (%). Descriptive statistics for the variables are shown on Table 2. We initially included all study participants. Only participants with missing data were removed from the analysis. Complete data were available for 12779 participants. Distribution histograms for these variables are shown on Fig 6A. For the multivariable analysis, we transformed the 10 Year Risk of CHD variable to make its distribution closer to normal (Fig 6A). Although distribution histograms are visually close to normal, none of them passed formal normality test. However, according to the central limit theorem, this requirement is waved for large sample size (greater than 100). [51]

**Table 2:** Basic descriptive statistics for the variables used in analysis of the association between sodium and 10 Years Risk of CHD in the ARIC Study (N = 12779).

	Mean	Median	Std Dev	5–95% Percentiles
Na^+^, mmol/l	141.0	141.0	2.4	137–145
Glucose, mmol/l	6.1	5.5	2.0	4.7–8.5
10 Years Risk of CHD, %	7.7	5.2	7.8	0.6–23.1
ln (10 Years Risk of CHD)	1.5	1.6	1.1	-0.5–3.1

In the multivariable model, containing serum sodium and blood glucose as independent variables both variables significantly (P<0.001) contribute to the predicted 10 Year Risk of CHD (F(2, 12776) = 598, P<0.001) (Fig 6B, Table 3). The positive regression coefficient for serum Na^+^ indicates that higher Na^+^ is accompanied by increased 10 Year Risk of CHD, consistent with overall findings of our study. Additionally, a three-dimensional plot of 10 Year Risk of CHD level vs. serum sodium concentration and age demonstrates that participants with higher concentrations of serum sodium have higher Risk of CHD at all ages (Fig 6C).

**Table 3:** Multivariable linear regression analysis of 10 Years Risk of CHD (Ln transformed) with serum Na^+^ and Glucose as predictor variables (ARIC Study).

			Predictive capability of independent variables						Analysis of variance	
Dependent variable:*Ln(10 Years Risk of CHD)*			Na^+^, mmol/l			Glucose, mmol/l				
		*Intercept*	*Coefficient*	*t*	*P*	*Coefficient*	*t*	*P*	*F*	*P*
All participants *(N = 12779)*		-1.86	0.017	4.2	<0.001	0.165	34.5	<0.001	598	<0.001
*No BP Medications *(N = 10336)*		-2.50	0.020	4.2	<0.001	0.176	29.4	<0.001	432	<0.001
BP Medications *(N = 3319)*		0.29	0.009	1.6	n.s.	0.082	16.4	<0.001	138	<0.001
*Glucose < 6.5 mmol/l *(N = 9360)*		-3.67	0.012	2.4	0.02	0.61	25.0	<0.001	319	<0.001
*White	Men *(N = 3788)*	-0.25	0.012	2.9	0.004	0.14	20.1	<0.001	202	<0.001
	Women *(N = 4453)*	-7.84	0.051	8.6	<0.001	0.23	24.8	<0.001	328	<0.001
*Black	Men *(N = 896)*	0.23	0.007	0.9	n.s.	0.09	12.8	<0.001	86	<0.001
	Women *(N = 1199)*	-4.14	0.027	2.7	0.006	0.15	17.0	<0.001	145	<0.001

Serum Na^+^ and glucose are significant predictors of the 10 Years Risk of CHD when all study participants are included in analysis and in the following subsets: people who do not take blood pressure medications, people with blood glucose concentration lower than 6.5 mmol/l, white men, white women and black men.

* Participants who took blood pressure medications were excluded from the analysis

To examine whether serum sodium and 10 years risk of CHD are still associated in different sub-groups within the study participants, we performed a similar analysis including only participants who took or did not take blood pressure medications, those with glucose level lower than 6.5 mmol/l and populations divided based on gender and race (Table 3). Serum sodium remained a significant predictor of CHD in people who did not take blood pressure medications but became insignificant in people who took blood pressure medication. Analysis of blood pressure and 10 years risk of CHD in these groups showed that people who took blood pressure medications still had significantly higher blood pressure and risk of CHD (118 ± 17 vs 131 ± 20 mm Hg (mean ± SD, P<0.001) and 6.6 ± 6.9 vs 12.0 ± 9.7% (mean ± SD, P<0.001)), suggesting that factors other than sodium become stronger predictors in people with higher blood pressure and higher risk of CHD. Therefore we excluded people who took blood pressure medications from further analysis. Serum sodium remained a significant predictor in people with blood glucose concentration lower than 6.5 mmol/l, white men, white women and black men. That serum sodium is a significant predictor in multiple subsets of the general population supports the general validity of the model. [51]

## Discussion

The present study addresses effects of small physiological elevations of extracellular sodium on the vascular endothelium and provides evidence supporting the hypothesis that pro-inflammatory changes produced by elevated sodium in endothelial cells might contribute to development of cardiovascular diseases.

In recent years experimental data have accumulated indicating that small alterations of extracellular sodium can produce changes in the physiology of endothelial cells. Normal plasma sodium concentration in the general population varies between 134 and 148 mmol/l, roughly corresponding to blood osmolality between 280 and 310 mosmol/kg. [13–15] During hypernatremia plasma sodium can reach 160 mmol/l (about 330 mosmol/kg) without clear symptoms (especially in elderly) and can be even higher during life threatening hypernatremia. [12] Systemic blood sodium concentration normally is closely regulated by renal variation of sodium and water excretion. Urinary concentration depends on maintenance of high sodium and osmolality in the renal medullary interstitial fluid. Depending on water and salt intake, osmolality in renal medullary interstitial fluid can reach 600 mosmol/kg in humans and 5000 mosmol/kg in rodents. The osmolality and electrolyte composition of extracellular fluid in other tissues had been believed to be similar to that of systemic blood because of rapid osmotic exchange across capillaries. However, recent studies have revealed exceptions. For example, when dietary salt intake is very high, some salt is temporarily and dynamically stored in the space between cells, bound to, for example, to glycosaminoglycans in the skin interstitium. [52, 53] The resulting skin microenvironment is hypertonic as evidenced by upregulation of the tonicity-responsive enhancer binding protein (TonEBP or NFAT5) in skin of rats on high salt diet, even though plasma sodium concentration has remained normal. [54] In another study, directly measuring osmolality of tissue slices, Go et al. demonstrated that osmolality of thymus, spleen and liver from normal mice is about 30 mosmol/kg higher than osmolality of the systemic blood. [55] Thus, local extracellular sodium concentration can be higher than in systemic blood plasma.

Even very small elevations of extracellular sodium produce physiologically relevant changes in endothelial cells. Thus, using atomic force microscopy, Oberleithner et al. demonstrated that acute (several minutes) increase of sodium from 135 to 145–150 mmol/l in presence of aldosterone stiffens endothelial cells in tissue culture and reduces nitric oxide release from them. [24, 25] Chronic (5 days) increase of sodium leads to hardening and flattering of the endothelial glycocalyx (eGC), decreased binding/buffering capacity of the eGC for sodium, and increased endothelial sodium permeability. [25]

The endothelial glycocalyx is a carbohydrate-rich layer lining the vascular endothelium. [26] Its composition is very dynamic. Various signals result in altered synthesis of its constantly turning over membrane bound molecules, which results in fine tuning of inflammatory responses. High NaCl-induced changes in composition of the eGC [25, 56] could involve alterations of cellular metabolism, biochemistry and/or signaling. Indeed, previous studies [27] and our present one indicate that small elevations of extracellular sodium increase expression of pro-inflammatory mediators in endothelial cells and increase their adhesive properties. Thus, Wild et al [27] demonstrated that short exposure (20 hours) of HUVEC cells in culture to elevated sodium (+10-30mmol/l) potentiated the pro-inflammatory effects of TNFα and of non-uniform shear stress. Their evidence included enhanced expression of adhesive molecules (VCAM-1 and E-selectin) and increased adhesion of monocytes to the endothelial cells. This effect was not seen under static or laminar flow conditions.[27] In the present study, we show that chronic exposure of HUVEC cells to elevated sodium for 4 days under static conditions increases expression of adhesive molecules (VCAM-1 and E-selectin) and of pro-inflammatory chemokine MCP-1 (CCL2), accompanied by increased adhesion of monocytes (Figs 1and 2). Production of such effects under static conditions, without additional pro-inflammatory stimulation by TNFα or non-uniform shear stress, suggests that elevated sodium per se can initiate inflammation.

Extrapolation of these findings suggested that elevation of plasma sodium for even a short time could enhance inflammation in arterial locations exposed to high shear stress such as curvatures, ostia and bifurcations, which are known to be prone to atherosclerosis. [57, 58] In addition, chronic elevation of plasma sodium or continuous high sodium in locations that accumulate it could cause inflammation as a result of the pro-inflammatory effects of sodium on the endothelium of the microvasculature. [59, 60] To test these possibilities in vivo we restricted water to elevate serum sodium in mice.

We chose water restriction, rather than high salt diet, as a way to increase extracellular sodium for the following reasons. First, water restriction increases serum sodium more efficiently than increasing salt intake. With the mild water restriction that we used, serum sodium increases by 5 mmol/l, without evident dehydration. [28] By contrast, in human studies, manipulation of salt intake within a physiologically relevant range (1-15g/day) change serum sodium concentration by only 2–3 mmol/l. [10, 61, 62], and the reported effects in rodents are even smaller, namely 1.5–2 mmol/l [54] or none at all [52] with salt added up to 8% of rodent diet [52, 54] The human equivalent of the latter is daily intake of about 40 g of salt (amount of salt in amount of rodent diet AIN-76A containing 2000 kcal). Thus, high salt diet-induced increase of serum sodium in mice is much less than can be achieved by physiologically realistic water restriction. A second confounding disadvantage of high salt diet is the likely increase of intravascular shear stress due to water retention and increased plasma volume.[63] This shear stress, itself, could independently increase intravascular inflammation [58, 64] Finally, we had already verified the water restriction model. [28] It not only increases serum sodium by 5 mmol/l, making it reasonable for studying the effects of high plasma sodium on atherosclerosis in large vessels, but it also increase hypertonic signaling in tissues, giving a plausible avenue for microvascular inflammation.

Consistent with the cell culture results, water restriction increased expression of mediators of inflammation VCAM-1, E-selectin and MCP-1 in mouse tissues (Fig 3). Also, water restriction promoted atherosclerosis in aortic root (Fig 4) and caused thickening of walls of coronary arteries in ApoE-^/-^ mice (Fig 5), which is considered as a risk factor for development of atherosclerosis and predicts future clinical cardiovascular disease events.[41, 42].

Although we attribute the vascular findings in our mouse model of water restriction to the increase of plasma sodium, other factors that changed during the water restriction could also be involved, such as activation of the renin-angiotensin-aldosterone system by water restriction [63]. An additional concern was that the mouse model might not translate to humans. Therefore, we analyzed the relation between plasma sodium and cardiovascular disease in humans, using the results of the Atherosclerosis Risk in Community (ARIC) study. [43]

The normal human population has much larger range of plasma sodium 135–145 mmol/l than was achieved in the experiments described above in which sodium was changed only 0–3 mmol/l by dietary salt manipulation and 5 mmol/l by manipulating water intake in mice. Therefore, the general human population represents a good model for testing the association of plasma sodium with other measured parameters of health. Using multivariable regression analysis [48–51] of data from ARIC study, we demonstrate that plasma sodium is an independent predictor for 10Years Risk for occurrence of coronary heart disease (CHD) (Fig 6, Tables 2 and 3). Such result is consistent with our proposed pro-inflammatory and pro-atherogenic effects of plasma sodium. Initially, it is difficult to comprehend how a single measurement of sodium on the first visit could predict future CHD if plasma sodium concentration is, as often viewed, a random value that varies normally due to assay-specific factors. However, plasma sodium has recently been shown to be an idiosyncratic characteristic of every person. This was shown through analysis of plasma sodium measurements in records from two large health plan-based cohorts over a 10 year interval.[29] Thus, serial plasma sodium values for an individual cluster around a patient-specific set point and the degree of individual variation is similar to that for glucose. The individuality of plasma sodium explains how a single measurement of plasma sodium could predict long-term health outcomes, such as we find in the 10 Years Risk of CHD in this study and 10 Years Risk of Stroke in our previous study. [28]

Taken together, the results of this and previous studies indicate that small elevations of extracellular sodium within or slightly above the normal plasma range have adverse cardiovascular consequences. Thus, in endothelial cell culture elevations of extracellular sodium increases endothelial stiffness; hardens and flattens the endothelial glycocalyx [24, 25]; increases expression of inflammatory mediators, such as adhesion molecules VCAM1 and E-selectin and chemokine MCP-1, accompanied by increased adhesive properties both during non-uniform shear stress and under static conditions ([27] and Figs 1 and 2); and increases expression and secretion of von Willebrand Factor, a key initiator of blood clotting [28]. In mice, mild water restriction that elevates plasma sodium by only 5 mmol/l upregulates vWF, the initiator of blood clotting in tissues and in blood [28]; upregulates the mediators of inflammation, VCAM-1, E-selectin and MCP-1 (Fig 3); increases thrombogenesis [28]; promotes atherosclerosis (Fig 4); and thickens the walls or coronary arteries (Fig 5). Also, multivariable analysis of data from Atherosclerosis Risk in Community study demonstrates that serum sodium is an independent predictor in humans of the plasma level of vWF, of the 10 Years Risk of Stroke [28] and of the10 Years Risk of CHD (Fig 6, Table 3).

In summary, the known mechanisms underlying the pathology of cardiovascular diseases such as atherosclerosis, CHD and stroke involve a complex interplay of inflammation, coagulation and vascular wall remodeling [19–21]. Now, there is increasing evidence that elevation of extracellular sodium within the physiological range is accompanied by vascular changes that facilitate development of CVD. The findings provide additional insight into the adverse cardiovascular effects of conditions affecting plasma sodium, such as dehydration, and other conditions affecting water/salt balance.[3–12] The findings also bring attention to plasma sodium as a possible risk factor for CVDs and probably for other diseases with thrombotic and inflammatory components.

## Methods

### Cell culture

Primary Umbilical Vein Endothelial Cells; Normal, Human (HUVECs) were purchased from American Type Culture Collection (ATCC) at passage 1 (ATCC PCS-100-010, ATCC, Manassas, VA). These cells are distributed by ATCC as part of the Primary Cell Solutions cell offerings. The cells were grown at 37C, 5% CO_2_ in Vascular Cell Basal Medium (No. PCS-100-030, ATCC) supplemented with Endothelial Cell Growth Kit-BBE (No.PCS-100-040, ATCC) and antibiotics (No.PCS-999-002, ATCC). Osmolality of this medium (Control medium) was 270 mosmol/kg (133 mmol/l sodium). High NaCl medium was prepared by adding NaCl to the total osmolality of 295–380 mosmol/kg (145–188 mmol/l sodium). To elevate NaCl, control medium was replaced by the high NaCl medium. All experiments, except transmigration assay, were performed on logarithmically growing cells at about 80% confluence. Completely confluent monolayers were used for transmigration assay. For all experiments, cells were exposed to high NaCl for 4 days. To prevent cultures from becoming over confluent, after 2 days in high NaCl medium cells were passaged to new dishes (RNA extraction), chamber slides (adhesion assay) or cell culture inserts (transmigration assay) and grown in high NaCl for 2 more days. Seeding cell number was adjusted so that they were about 80% confluent at time of sample collection for mRNA analysis and adhesion assay or completely confluent for transmigration assay. For all experiments, cells were used at passages P2-P6.

### Analysis of effect of high NaCl on gene expression in HUVECs by PCR array

Cells were exposed to high NaCl for 4 days on 10 cm dishes. The Human Endothelial Cell Biology RT² *Profiler* PCR Array (Cat No 330231 PAHS-015ZA, Qiagen, Valencia, CA) was used to analyze the expression of 84 genes related to endothelial cell biology. Total RNA was extracted with RNeasy Mini Kit (No. 74104, QIAGEN, Valencia, CA). 0.5 μg of the RNA was converted into cDNA using RT^2^ First Strand Kit (Cat No 330401, Qiagen, Valencia, CA). Quantification of the genes expression was performed by the Comparative C_T_ method. B2M, HPRT1, GAPDH, ACTB housekeeping genes (HKGs) were used as an endogenous controls. Average C_T_ values for these four HKGs (AVG HKGs) were used for the quantification. The relative copy number (2 –^(Ct (mRNA)—Ct (AVG HKGs)^) of each target mRNA was calculated for each sample and normalized to the relative copy number in the corresponding control sample (270 mosmol/kg). The experiment was repeated 3 times.

### Immunofluorescent detection of E-selectin in HUVEC cells

Cells grown on 8 chamber slides were fixed for 10 minutes in 2% formaldehyde (No. 18814, Polysciences, Inc, Warrington, PA) at room temperature, followed by 10 min fixation in 100% methanol at -20C. The cells were washed 3 times with 0.1% Triton X-100 in PBS and blocked with 3% bovine serum albumin for 1 hour at room temperature. Slides were incubated with primary antibodies for E-selectin (ECM645, Part No. 2004183, Chemicon, Billerica, Massachusetts) at 4°C overnight, followed by secondary antibodies labeled with Alexa Fluor 488 nm (green emission) (No. 4408, Cell Signaling, Danvers, MA) at room temperature for 1 hour. After 2 washes with PBS, cells were stained with 2.5μg/ml DAPI (DNA stain) (No. D1306, Invitrogen, Carlsbad, CA) and mounted with ProLong Gold antifade reagent (No. P36930, Invitrogen, Carlsbad, CA). Pictures were taken by confocal microscopy using a Zeiss LSM 510 microscope (Carl Zeiss MicroImaging, Jena, Germany) with a 63× NA1.4 oil-immersion objective.

### Extraction of protein from HUVECs and Western blot

Cells were rinsed with phosphate-buffered saline (PBS), adjusted with NaCl to the same osmolality as the medium, then lysed with RIPA buffer (50 mM Tris-HCl, 1% NP-40, 150mM NaCl, 1mM EDTA, 1mM NaF, 1 mM Na_3_VO_4_, and protease inhibitors (Roche Diagnostics, Indianapolis, IN)). 3X reducing Laemmli Sample buffer (No. 7722, Cell Signaling, Danvers, MA) was added to the lysates and samples were boiled for 5 minutes. Sample loading onto gels was equalized according to the total protein concentration measured before addition of Laemmli buffer. Primary antibodies were against VCAM-1 (No. 305801, BioLegend, San Diego, CA), alfa-tubulin (No. 691251, MP Biomedicals, Solon, OH). Secondary antibodies were labelled with Alexa Fluor 680 nm Dye (Invitrogen, Carlsbad, CA). Immunoblots were scanned and Integral Fluorescence (IF) from each band was measured using Odyssey Infrared Imaging System (Li-COR Biosciences, Lincoln, NE).

### Adhesion assay

Adhesion assay was performed using Endothelial Cell Adhesion Assay Kit (Cat No: ECM645, Chemicon, Billerica, Massachusetts) according to the kit instructions with modifications. HUVECs were exposed to high NaCl for 4 days as described above in “Cell culture” section. For the last 2 days of the exposure they were grown on 8 chamber slides where adhesion assay was performed. Fresh adult human peripheral blood mononuclear cells (PBMC) (Cat No: PB009F-1, HemaCare, Van Nuys, CA) were frozen in liquid nitrogen in 2.5 mln aliquots in FBS with 20% DMSO. For the adhesion assay, PBMC were thawed, transferred into HUVEC cell culture media described above and incubated overnight at 37C, 5% CO_2_. Then PBMC were labeled with Calcein AM Dye: PBMC were pelleted at 240 g for 10 min, re-suspended in 1 ml culture medium with 2.5 uM of Calcein AM from the kit and incubated at 37C, 5% CO_2_ for 30 min. PBMC were then washed 3 times with HUVEC media and added to HUVEC cells (150,000 labeled PBMC per chamber). PBMC were allowed to settle down and adhere to HUVECs for 1h. Then unbound PBMC were washed out by gentle removal and addition of culture medium in chambers for 3 times. Following the washing of unbound PBMC, HUVECs with attached PBMC were fixed for 10 minutes in 2% formaldehyde (No. 18814, Polysciences, Inc, Warrington, PA) at room temperature, permeabilyzed with 0.1% Triton X-100, stained with 2.5μg/ml DAPI (DNA stain) (No. D1306, Invitrogen, Carlsbad, CA) and mounted with ProLong Gold antifade reagent (No. P36930, Invitrogen, Carlsbad, CA) (Fig 2D). To quantify the number of PBMC attached to HUVECs, the slides were scanned with Laser Scanning Cytometer (CompuCyte, Cambridge, MA). PBMC were identified and counted based on Calcein AM green fluorescence (excitation 495nm / emission 516 nm) and total number of cells was counted based on DAPI fluorescence (350 nm excitation / 470 nm emission). It is important to note that before the addition of PBMC, cell culture medium in all chambers were changed to control culture medium (270 mosmol/kg). Therefore, this assay tests changes of adhesive properties of endothelial cells following exposure to high NaCl and excludes effects of high NaCl on PBMC themselves. For positive control, HUVECs were treated for 5h wth TNFα (50 ng/ml), a known inducer of adhesive properties of endothelial cells.

### Transmigration assay

To analyze whether exposure of HUVECs to high NaCl promotes transmigration of PBMC through HUVECs monolayer, transmigration assay was performed [65]. HUVECs were exposed to high NaCl for 4 days as described above in “Cell culture” section. For the last 2 days of the exposure they were seeded in fibronectin coated cell culture inserts for 24 well plates with 3.0 μm pores (No. 354597, Corning, Tewksbury, MA). Cell density was adjusted to ensure that confluent monolayers were formed by the end of 4 days exposure to high NaCl (100,000 HUVECs per insert in 250ul). PBMC were prepared and labeled with Calcein AM the same way as described above for adhesion assay. 250,000 labeled PBMC in 250 ul of cell culture medium (270 mosmol/kg) were added to the HUVEC monolayer, the cell culture inserts were placed into wells of 24 well plate filled with 300 ul of cell culture medium (270 mosmol/kg) and incubated at 37C, 5% CO_2_ 6h. To quantify number of PBMC transmigrated through HUVECs monolayers from upper chamber to lower chamber, culture media from lower chamber was transferred into wells of 96 well plate and integral green fluorescence was measured by Wallac 1420 Victor multilabel counter (PerkinElmer, WalthamMA). Note that as in case of adhesion assay, high NaCl was washed out from HUVEC monolayers before addition of PBMC. Therefore, the assay tests effects of high NaCl on ability of HUVECs to facilitate PBMC transmigration but excludes effects of high NaCl on PBMC themselves.

### Mice

There were two mouse models used in this study. Male 129S6 traditional inbred mice (129S6, Model no.129SVE-M, Taconic Farms, Inc, Hudson, NY) were purchased at age of 3 months and used for 9 days water restriction experiment. Male ApoE constitutive knockout mice (Apoe, Model no.APOE-M, C57BL/6 Background), Taconic Farms, Inc, Hudson, NY) were purchased at age of 4 weeks. All mouse studies were carried out in strict accordance with the recommendations in the Guide for the Care and Use of Laboratory Animals of the National Institutes of Health. The protocol was approved by the Animal Care and Use Committee of National Heart Lung and Blood Institute (Protocol Number: H-0130R2). The mice were housed in an Association for Assessment and Accreditation of Laboratory Animal Care-accredited facility of National Heart Lung and Blood Institute. At the end of the experiments, mice were sacrificed by cervical dislocation.

### Water restriction (9 days)

Water restriction was performed as previously described [28]. All mice received gelled food containing 30% water (1.7 ml of deionized water + 4 g of balanced purified rodent diet, AIN-76A, Research Diets, New Brunswick, NJ + 57 mg of agar per 5.7 g of the food). Food was provided in excess in individual cups so the mice ate what they wanted. The control group had free access to water. Water restricted mice did not get any additional water. Five control and five water restricted mice were tested.

### Mouse blood collection and measurement of cholesterol in mouse serum

Blood was collected from the tails of the mice. To prepare serum, the blood was allowed to clot by leaving it undisturbed at room temperature for 30 min, and then the clot was removed by centrifuging at 4°C for 10 min at 1800g. The Amplex Red Cholesterol Assay Kit (No. A12216, Life Technologies) was used to measure serum cholesterol level.

### RNA extraction from mouse tissues and analysis by real-time PCR

Tissues were collected in Allprotect Tissue Reagent (No. 76405, QIAGEN, Valencia, CA) at the end of the experiments and stored at -20°C. Total RNA was extracted using AllPrep DNA/RNA Mini Kit (No. 80204, QIAGEN, Valencia, CA). The RNA (1 μg) was converted to cDNA by reverse transcription using TaqMan High-capacity cDNA RT Kit (No. 4374966, Applied Biosystems). VCAM-1, E-selectin and MCP-1 mRNA levels were measured by real-time PCR using Mouse TaqMan Gene Expression Assays (No. 4331182, Applied Biosystems) on 7900HT Fast Real-Time PCR System (Applied Biosystems). Quantification was performed by the Comparative CT method. 18S ribosomal RNA was used as an endogenous control (No. 4319413E, Applied Biosystems) and was amplified in the same tube with the target. The relative copy number (2 –^(Ct (vWF)—Ct (18S rRNA)^) of a target was calculated for each sample and normalized to the average copy number in the corresponding tissues of control mice (free access to water). Each group contained 5 mice.

### Immunohistochemical staining of paraffin embedded mouse liver sections

Mouse livers were fixed for 48 hours in 4% paraformaldehyde at 4°C, and then embedded in paraffin. Sections were cut and mounted on positively charged slides by American Histolabs (Gaithersburg, MD). Sections were stained with antibodies against VCAM-1 (No. MCA2297T, AbDSerotec, Kidlington, UK), CD31 (No. DIA 310, Dianova, Hamburg, Germany), as previously described [66]. Visualization was with diaminobenzidine (DAB) (No. D22187, Molecular Probes, Eugene, OR), which generates a brown-colored oxidation product upon reaction with the horseradish peroxidase (HRP) labeled secondary antibody. Development of the brown DAB color was monitored to prevent over saturation that would prevent quantification. A Nikon E800 Widefield Microscope was used for photography.

### Quantification of VCAM-1 protein expression on mouse liver tissue sections

The quantification was performed as previously described [28]. Briefly, to quantify VCAM-1 protein expression on the DAB stained sections (see above), we used the Yellow-CMYK channel method described by Pham *et al* [67]. They demonstrated that images in the CMYK yellow channel correlate with the IHC DAB stain intensity. We extracted the CMYK yellow channel from the images of liver tissue sections stained with DAB for VCAM-1 (brown color) and with hematoxylin for nuclei (blue) (Fig 3B and 3C) and quantified average yellow intensity per pixel as a measure of VCAM-1 protein expression. Images of liver sections from 4 control and 4 water restricted mice were analyzed. 20 images were taken from the liver section of each mouse and their average intensity was calculated.

### Quantification of atherosclerosis in aortic root and wall thickness of coronary arteries of ApoE^-/-^ mice

Atherosclerotic lesions in aortic root were quantified as described [68] with slight modifications. Starting at age of 6 weeks, ApoE^-/-^ mice were water restricted as described above by feeding them gel food containing 30% water (Fig 4A). Gel food was made from high fat RD Western Diet (D12079BM, Research Diets, New Brunswick), containing 40% of calories from fat. Blood for cholesterol measurements was collected 6 weeks after start of the water restriction. Atherosclerosis was analyzed 7–9 weeks after start of the water restriction. Heart/aortic root tissues were fixed in 4% formaldehyde (No. 18814, Polysciences, Inc, Warrington, PA) for 2 days at 4C.

Upper part of the hearts containing aortic root were transferred into PBS and then embedded in O.C.T. compound (Tissue-Tek #4583, Sakura Finetek, Torrance, CA). Serial 10-μm frozen sections were cut with Leica CM3050 cryostat microtome (Leica Biosystems) spanning 900 μm starting from the base of the aortic root. Every other section was picked up onto glass slides, dried at room temperature for 10 min and then stored at -80C. For atherosclerotic lesions analysis, lipids were stained with Oil Red O (No.26503-02, Electron Microscopy Sciences, Hatfield, PA) and stained area on every section was measured with Fiji image processing software [69].

Lower parts of the hearts were embedded in paraffin. Sections were cut and mounted on positively charged slides and stained with hematoxylin and eosin by American Histolabs (Gaithersburg, MD). Images of coronary arteries were taken by Nikon E800 Widefield Microscope from 3 control and 4 water restricted mice and analyzed with Fiji image processing software [69]. Arterial lumen diameter was calculated from inner wall perimeter. Arterial wall thickness was calculated as average of 8 measurements along the wall of each artery.

### Analysis of data from Atherosclerosis Risk in Community (ARIC) study

Data from Atherosclerosis Risk in Community (ARIC) study were obtained from the NHLBI Biologic Specimen and Data Repository Information Coordinating Center. The obtained datasets were redacted to remove personal identifiers to conform to individual informed consent restrictions. The datasets transfer was approved by NIH Office of Human Subjects Research (OHSR) and excluded from Institutional Review Board review due to the determination of Not Human Subjects Research that was based on the interpretation of 45 CRF 46 under “Research Involving Coded Private Information or Biological Specimens” and Guidance on Engagement of Institutions in Human Subjects Research (October 16, 2008).

Multivariable linear regression analysis [48, 50, 51] of data from ARIC study was performed to assay if concentration of serum sodium contributes to prediction of 10 years Risk of coronary heart disease (CHD). The data were analyzed using SAS (SAS Institute Inc., Cary, NC, USA) and SigmaPlot (Systat Software, San Jose, CA) software.

### Statistical analysis

All the statistical analyses were done using SigmaPlot Software. All data were tested for normality and equal variance before the analysis. If normality and equal variance tests passed, comparisons were performed by the two-tailed Student's *t*-test. If not, non-parametric Mann-Whitney Test was used. *P*<0.05 was considered significant. VCAM-1 expression data (Fig 1B) were Log10 transformed before the analysis. VCAM-1 protein level in HUVEC cells was analyzed for linear dependence on the level of NaCl using GraphPad InStat Software.

## Supporting Information

S1 Fig Representative images from immunohistochemical staining for VCAM-1 protein of heart sections. Positive staining of inner surface of coronary arteries of water restricted mice (shown by arrowheads) is consistent with endothelial expression of VCAM1.
(TIF)
