# Supplementary figures and images for: Elevated Sodium and Dehydration Stimulate Inflammatory Signaling in Endothelial Cells and Promote Atherosclerosis
Source: PLoS One. 2015 Jun 4;10(6):e0128870. doi: 10.1371/journal.pone.0128870 (PMC4456159; doi:10.1371/journal.pone.0128870)

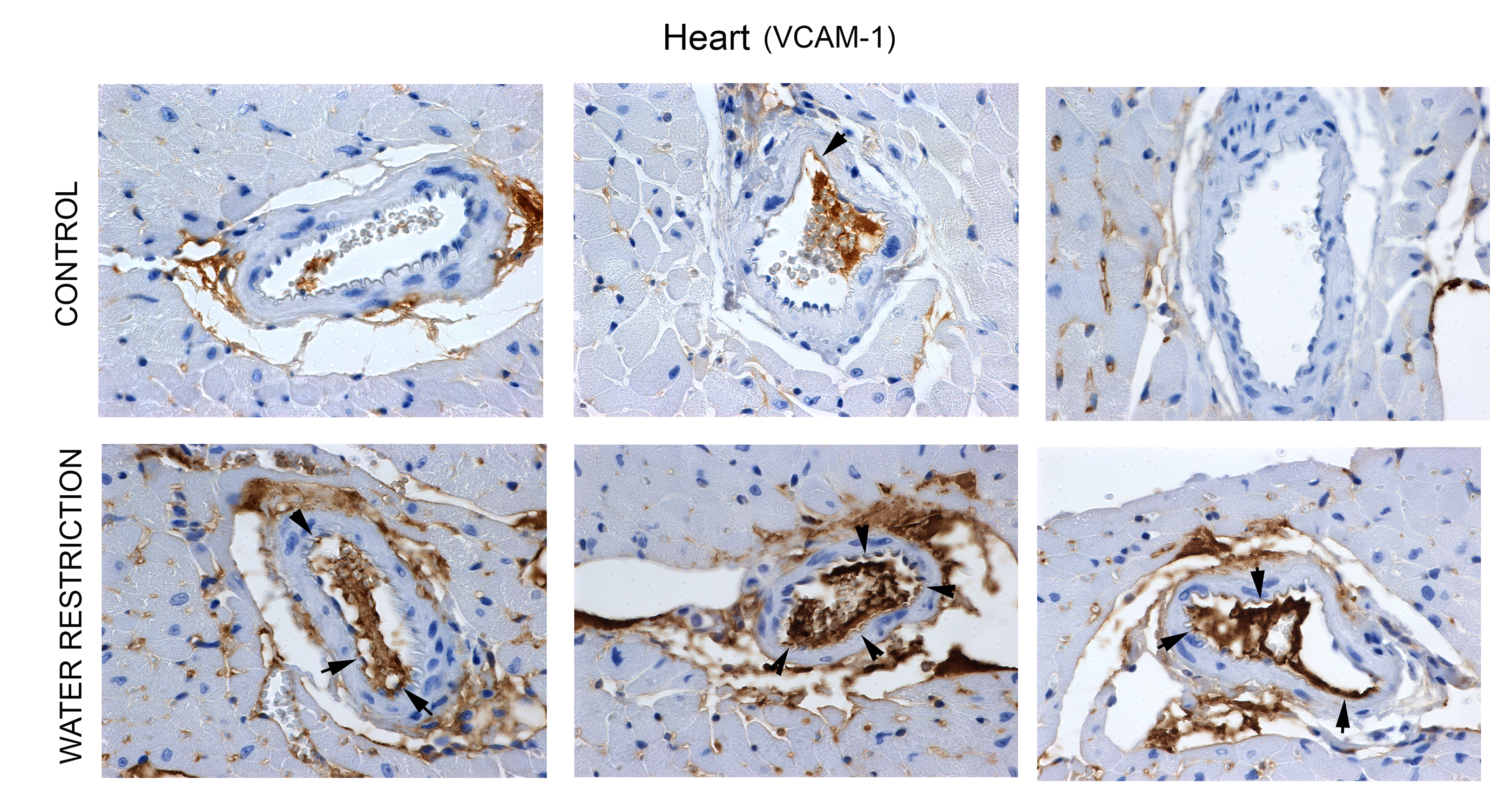

Supplement: S1 Fig — Positive staining of inner surface of coronary arteries of water restricted mice (shown by arrowheads) is consistent with endothelial expression of VCAM1. (TIF) [file pone.0128870.s001.tif]
